# Successful fertility following optimized perfusion and cryopreservation of whole ovary and allotransplantation in a premature ovarian insufficiency rat model

**DOI:** 10.1186/s13048-018-0401-4

**Published:** 2018-05-02

**Authors:** Yan Ding, Jia-liang Shao, Jun-wei Li, Ying Zhang, Kai-hua Hong, Ke-qin Hua, Xiang Wang

**Affiliations:** 1 Department of Gynecology, Obstetrics and Gynecology Hospital, Fudan University, Shanghai, 200090 China; 2 Department of Urology, Shanghai General Hospital, Shanghai Jiao Tong University School of Medicine, Shanghai, 200080 China

**Keywords:** Whole ovaries, Perfusion, Cryopreservation, Fertility restoration, Premature ovarian insufficiency

## Abstract

**Background:**

Fertility preservation by whole ovary cryopreservation and transplantation (WOCP&TP) with vascular anastomosis requires successful cryopreservation. In this study, we investigated the possibility of restoring ovarian function and natural fertility after WOCP&TP in a premature ovarian insufficiency (POI) rat model. The influence of cryopreservation on the offspring of rats following WOCP&TP was also explored.

**Method:**

Rats aged 8-10 weeks were used as donors and recipients for allotransplantation. Fifteen rat whole ovaries were divided into three groups: the optimized group, the conventional group, and the fresh group. Different perfusion modes were used before cryopreservation and after thawing. Whole ovaries were observed by morphologic analysis, immunohistochemical staining, and transferase-mediated deoxyuridine triphosphate nick end-labeling assay. Ovarian function and fertility after WOCP&TP were then observed in 25 cyclophosphamide-induced POI rats for 8 months. Ovarian function was assessed by vaginal smears and blood hormone levels. Fertility restoration was quantified as the live birth rate after mating. The filial generation of rats was mated at 8-10 weeks of age. Offspring were observed for birth defect.

**Results:**

Histological evaluation demonstrated intact morphology of follicles in all groups, with 77.6% of the total number of follicles identified as intact in the optimized group. The apoptotic rates of ovarian cells in the optimized group were significantly lower than that in the conventional group. Of the 20 live POI rats, 14 (70%) began to recover ovarian function after 2 weeks of transplantation, with normal hormone levels achieved 4 weeks after transplantation. Four of 14 rats were pregnant and delivered live offspring. One rat had a second pregnancy and delivered a second litter of live offspring. When the offspring matured, they were mated, and second and third generations of rats were born. All offspring had no abnormalities in appearance.

**Conclusions:**

High rates of restoration of ovarian function and natural fertility with multiple generations of offspring were obtained following WOCP&TP in a cyclophosphamide-induced POI rat model by utilizing optimized perfusion. Cryopreservation did not affect the viability of successive generations.

## Background

As the proportion of survivors of childhood cancer and of cancer during the reproductive years increases, the quality of life after cancer treatment has become an important consideration. Among the toxic side effects of chemotherapy and radiotherapy, one of the greatest concerns for young women is the risk of sterility or premature ovarian insufficiency (POI).

A number of strategies for fertility preservation have been developed in recent years. Human ovarian cortical tissue cryopreservation and autotransplantation represents a potentially valuable technique for the preservation and restoration of fertility. To date, more than 86 babies have been born worldwide as a result of transplanted ovarian tissue [1–3]. However, the transplanted fragments of ovarian tissue contain only a fraction of an individual’s ovarian reserve. The problem of graft longevity is exacerbated by freeze-thaw damage and high rates of follicle loss due to ischemia during revascularization. In contrast, whole ovarian cryopreservation and transplantation (WOCP&TP) with vascular reanastomosis represents a potentially superior alternative as this technique not only allows the preservation and transfer of the entire follicle reserve, but also allows complete and immediate revascularization and thus the potential for complete restoration of ovarian function.

In theory, whole ovarian cryopreservation is attractive. In fact, successful cryopreservation and transplantation of whole ovaries has been reported in different experimental animal species (mice, rat, rabbit, dog, and sheep) [4]. However, the increased mass, complexity, and diversity of tissues being cryopreserved raises a number of technical issues. One of these initial problems was the ability to fully permeate the organ with cryoprotective agents (CPA) such as dimethyl sulphoxide (DMSO). This was subsequently solved by cannulating the ovary via the ovarian artery and perfusing it with the cryoprotective media [5]. Studies showed that both perfusion and cryopreservation may have deleterious effects on the ovary, in particular on the ovarian microvasculature within the ovarian medulla, which could result in substantial follicle loss [6–9]. Therefore, attempts to perform WOCP&TP have met with limited success. In the present experiment, we report an optimized perfusion apparatus in which whole ovaries were perfused in a mode of a linearly increasing DMSO concentration at a constant speed before cryopreservation. A rat model of POI induced by cyclophosphamide (CTX) was established to simulate female patients with cancer who had been treated with chemotherapy. The optimization of perfusion following WOCP&TP in the POI model led to high rates of restoration of ovarian function and natural fertility with multiple generations of live births.

## Results

### Morphologic assessment of whole ovary after cryopreservation and thawing

**Fig. 1:**
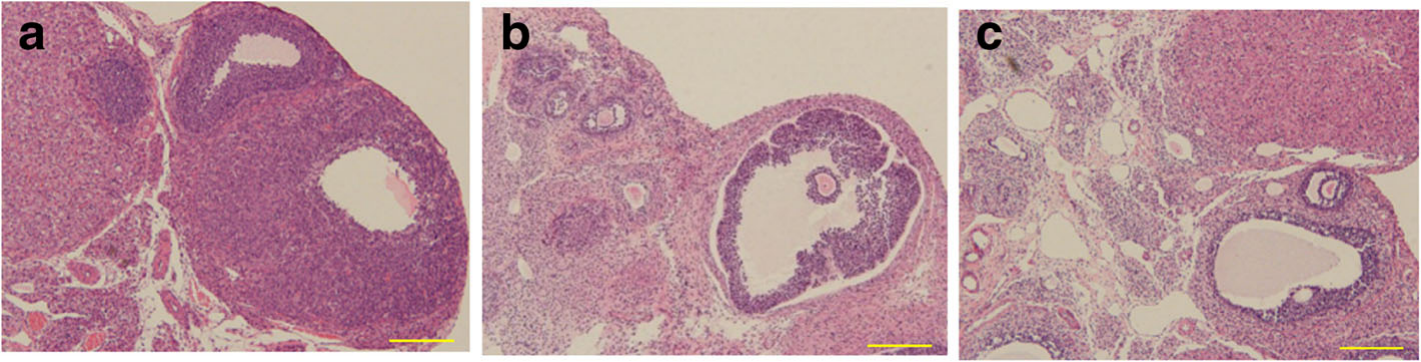
The morphological changes of the ovary in the different groups. Representative histologic images of ovary stained by hematoxylin and eosin in the fresh group (**a**), conventional group (**b**) and optimized group (**c**). The ovary in the optimized group showed well-preserved general morphology of the follicles. (scale bar =100 μm)

**Fig. 2:**
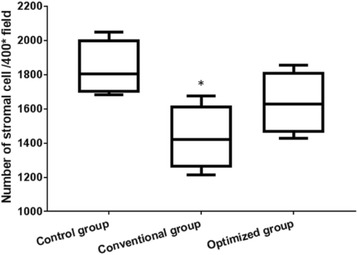
Quantitative analysis of ovarian stromal cells in the different groups. Compared with the control group, *P < 0.05

Histological evaluation and comparison of fresh ovaries versus cryopreserved/thawed ovaries demonstrated a well-preserved general morphology of the follicles in the optimized group, although there were areas of decreased stromal density or oocyte cytoplasm vacuolations in some samples (Fig. 1). However, more abnormal follicles could be seen in the conventional group. Data for the follicles at all developmental stages are reported in Table 1. The number of follicles in the primordial and primary follicle classes significantly decreased in the optimized group compared with the fresh group (*P* < 0.05). There was no significant difference in the number of secondary follicles and antral follicles between the two groups. In addition, 77.6% of the total follicles were counted in the optimized group, which was significantly higher than that of the conventional group (*P* = 0.002), although this value was still significantly reduced compared with the fresh group. The quantitative analysis of the stromal cells showed that there was no significant difference between the optimized group and the control group (*P* > 0.05) (Fig. 2).

**Table 1:** Follicles in representative sections of three groups

Groups	Number of follicles				Total number of follicles
	primordial	primary	secondary	antral	
Optimized group (*n* = 5)	79.8 ± 7.0^a^	22.5 ± 2.1^a^	9.8 ± 1.0^a^	3.8 ± 1.0^a^	118.0 ± 6.9^a^ (77.6%)
Conventional group (*n* = 5)	66.3 ± 5.9^a^	17.0 ± 1.8^a^	7.5 ± 1.3^a^	3.0 ± 0.8^a^	94.5 ± 7.3^a^ (61.0%)
Fresh group (*n* = 5)	104.8 ± 6.7	28.3 ± 3.5	12.5 ± 1.7	7.0 ± 0.8	152.5 ± 11.6 (100%)

^a^Compared with the fresh group, *P* < 0.05

### Apoptosis analysis

**Fig. 3:**
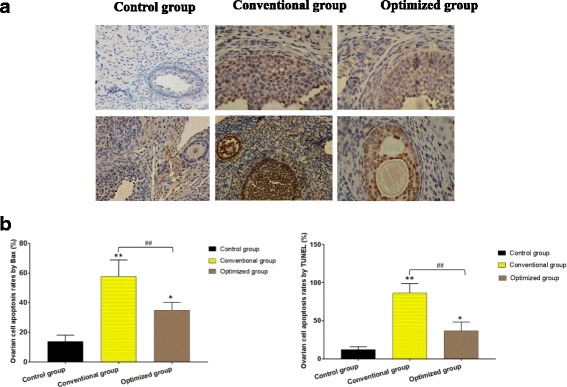
Apoptosis of ovarian cells in the different groups. **a** Representative ovarian images by Bax and TUNEL staining in the fresh group, conventional group and optimized group. (scale bar =50 μm). **b** The apoptosis rates of ovarian cells in different groups compared with the control group, *P < 0.05, **P < 0.01; compared with the conventional group, ^##^*P* < 0.01

TUNEL-positive cells and Bax-positive cells were detected in the three groups, and representative images are shown in Fig. 3. The results were similar in the TUNEL and Bax analyses. Very few positive staining signals were detected in the fresh group. Positive staining signals in the conventional group were found in every stage of the follicles and the stromal cells, which were significantly increased in number compared with the fresh group (*P* < 0.01). While positive staining signals in the optimized group were found mostly in the antral follicles, they were significantly reduced in number compared with the conventional group (*P* < 0.01).

### Establishment of POI rats

All 25 rats treated with CTX survived. The weight of the rats slightly decreased during CTX treatment but recovered after 2 weeks of CTX treatment. The rats showed abnormal estrus cycles 7-10 days after CTX injection, with prolonged and irregular diestrum or absence of preoestrus and estrus. The estrus cycles of treated rats showed an average duration of 8.00 ± 2.83 days, and were still disordered after 2 weeks of CTX treatment; this duration was significantly longer than that of the control group (3.98 ± 0.85 days; *P* < 0.05). After 2 weeks of CTX treatment, serum FSH levels in the POI group were significantly higher than the control group (*P* < 0.01). Serum estradiol-17β and progesterone levels in the POI group were significantly lower than the control group after 2 weeks of CTX treatment (*P* < 0.01) (Table 2). Contracted ovaries and uteruses were clearly present in the POI group when the abdomen was surgically opened for transplantation.

**Table 2:** Serum hormone levels in rats after 2 weeks of CTX treatment

Groups	FSH (IU/L)	estradiol-17β (ng/L)	progesterone (pmol/L)
POI group (*n* = 20)	21.97 ± 1.85^a^	54.23 ± 5.71^a^	1315.25 ± 67.52^a^
control group (*n* = 20)	13.31 ± 0.75	80.84 ± 0.89	1689.36 ± 66.36

^a^compared with the control group, *P* < 0.01

*FSH* Follicle stimulation hormone, *POI* Premature ovarian insufficiency

### Outcome of transplantation

The median duration of transplantation surgery from incision to closure was 54.00 ± 9.64 min, and the estimated blood loss was < 1 ml in all animals. In all 25 allotransplanted rats, recirculation of the graft was satisfactory based on a color change from white to reddish, with pulsation through the anastomosed vessels. The weight of the rats began to increase after 4 days of significant reduction. Five rats died within 3 days after transplantation due to infection or anastomotic stenosis with thrombosis, while the remaining 20 rats survived after transplantation.

### Function assessment in vivo

**Fig. 4:**
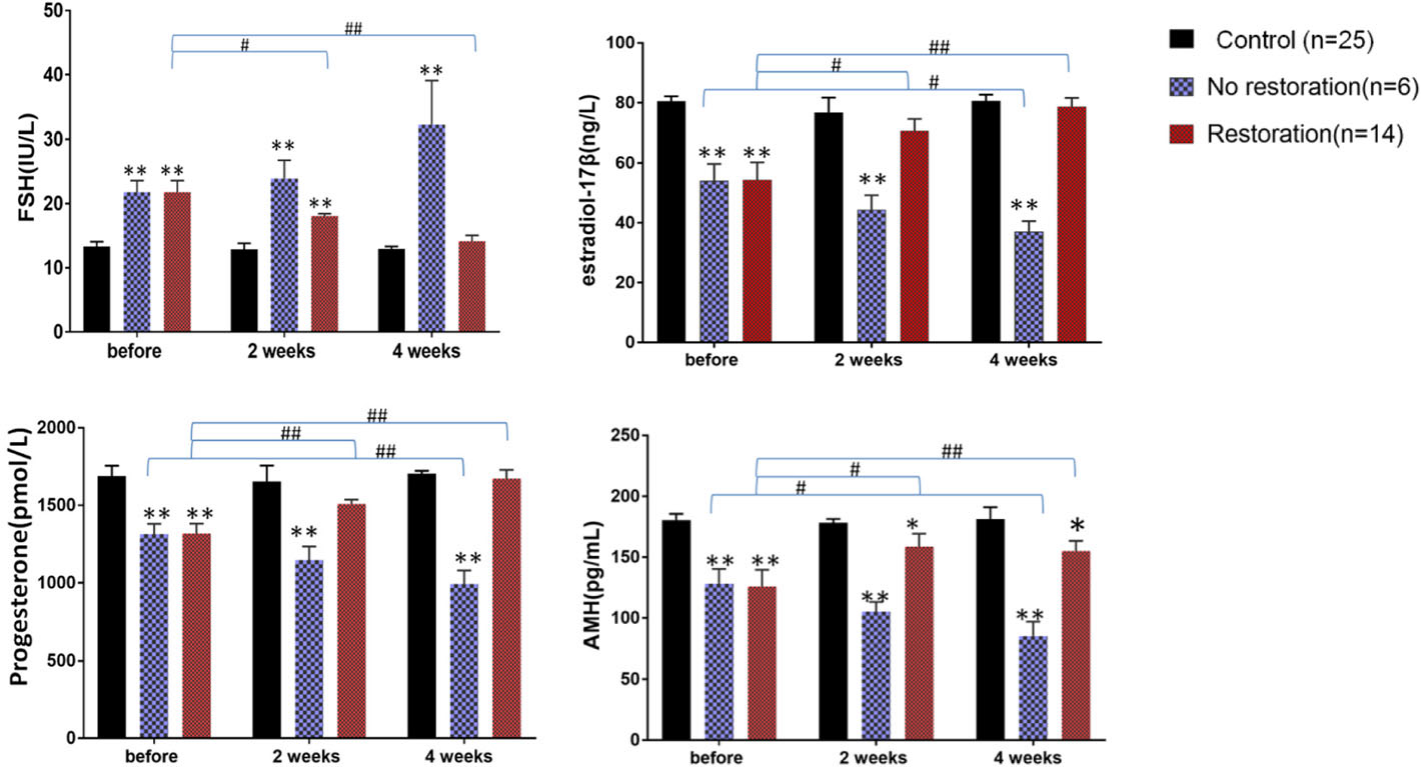
Changes in hormone levels after WOCP&TP in the different groups. Serum hormone levels after whole ovarian cryopreservation and transplantation in a premature ovarian insufficiency rat model. Compared with the control group, *P < 0.05, **P < 0.01; compared with before transplantation, ^#^*P* < 0.05, ^##^*P* < 0.01

Of the 20 live rats, 14 (70%) began to recover estrous cycles an average of 14.43 ± 2.98 days after WOCP&TP according to vaginal smears. Normal estrous cycles (4.65 ± 1.63 days) were seen 4 weeks after transplantation (*P* > 0.05). Similarly, serum levels of FSH significantly declined 2 weeks after transplantation (*P* < 0.05), but were still higher than levels in the control group (*P* < 0.01). Four weeks after transplantation, the serum levels of FSH continued to decrease and returned to normal levels (*P* > 0.05). Serum levels of progesterone and estradiol-17β significantly increased to normal levels 2 weeks after transplantation (*P* > 0.05). Serum levels of AMH significantly increased 2 weeks after transplantation (*P* < 0.05), but were still lower than levels in the control group (*P* < 0.05). Four weeks after transplantation, serum levels of AMH continued to increase and returned to normal levels (*P* > 0.05) (Fig. 4). The normal hormone levels of rats were maintained until they were sacrificed.

**Fig. 5:**
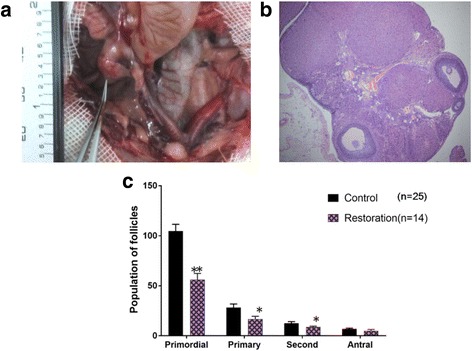
The transplanted ovary after 8 months of WOCP&TP. **a** Illustration of the premature ovarian insufficiency rat that became pregnant twice after whole ovary cryopreservation and transplantation (WOCP&TP). The right ovary and uterus appeared normal after 8 months of WOCP&TP with an absent left ovary. The right uterus was the same size as the contralateral uterus. **b** Histological image in an ovary recovered 8 months after WOCP&TP. There were all developing stages of follicles and corpora lutea in the right ovary. Scale bar = 200 μm. **c** Number of ovary follicles in the rats that resumed estrous cycles after WOCP&TP

The ovaries of the four rats that became pregnant were of normal appearance and vascularity and contained morphologically normal, well vascularized corpora lutea and all developmental stages of follicles. As seen in Fig. 5**,** the transplanted right ovary appeared normal 8 months after WOCP&TP, with the left ovary absent in the rat that developed a second pregnancy. There were follicles from primordial to antral stages and corpora lutea in the right ovary. The right transplanted uterine segment was the same size as the contralateral uterine segment. The average weight of the ovaries in the four rats that became pregnant was 25.84 mg, comparable with that of the control group (25.79 mg; *P* > 0.05). The ovaries of the 10 rats that did not become pregnant were of normal appearance in three rats and showed adhesions in seven rats. The average weight of the ovaries in the 10 rats that did not become pregnant was 22.84 mg, which was not different from that of the control group (*P* > 0.05). However, the number of primordial and pre-antral follicles in the 14 rats that resumed estrous cycles was significantly reduced compared with that of the control group (*P* < 0.05).

The remaining six rats showed a total absence of all cells on the vaginal smear, revealing no evidence of ovarian function restoration following WOCP&TP. These rats had significantly higher levels of FSH and lower levels of progesterone, estradiol-17β, and AMH at the first month; after 1 month, levels of progesterone, estradiol-17β, and AMH were maintained or continued to decline to being undetectable (*P* < 0.01) (Fig. 4). The transplanted ovaries and vascular pedicle in these non-restored rats were atrophic or absent with severe adhesions at the time of postmortem examination, and the weight of the ovaries could not be calculated.

### Fertility and offspring

**Fig. 6:**
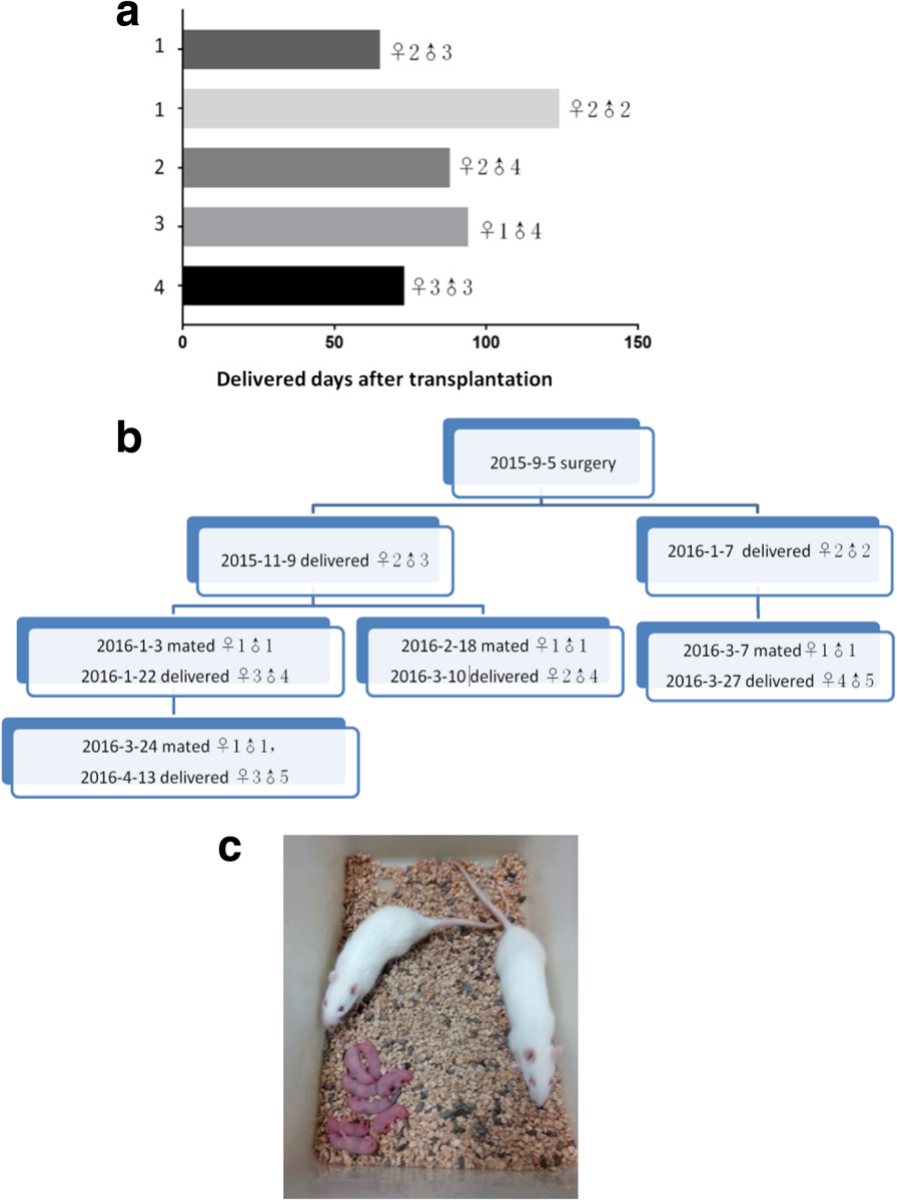
Fertility in rats after WOCP&TP. **a** Diagram summarizing the results of the rats that delivered births and the gender of offspring. Bars represent time point of delivering in days after WOCP&TP. **b** Family tree of the premature ovarian insufficiency rat that had three generations of offspring. **c** Image of rats with the third generation of offspring on April 13, 2016

Fourteen rats in the POI group that showed recovery of hormone levels were mated after 1 month of WOCP&TP. Four rats (20%) were pregnant within 2 months after mating and all delivered healthy live births. The total number of offspring was 22, and the number of offspring per litter was ranged from 4 to 6, which was statistically less than the normal yield of 8-13 (*P* < 0.05). A summary of the time of delivery and gender of offspring is presented in Fig. 6a. One month after delivery, one of the four rats underwent a second mating, became pregnant, and delivered a second litter of four live offspring. The first filial generation of rats was mated at 8-10 weeks age. They gave live birth to the second generation of offspring. In the same way, two rats of the second generation were mated when they were 8-10 weeks old, and the third generation of offspring was delivered. A family tree of the mated rats that produced three generations of offspring is shown in Fig. 6b. The number of live births in the filial generations ranged from 6 to 9, with no statistical difference compared with the normal yield (*P* > 0.05). All offspring had no abnormalities in appearance (Fig. 6c).

## Discussion

Transplantation of whole ovaries with vascular anastomosis has been considered to be a promising strategy for restoration of ovarian function and fertility in cancer patients. Until now, five reports have described grafts of fresh whole ovary with vascular anastomosis in humans, and one successful pregnancy was recorded [10–14]. In the past few years, attempts at cryopreservation and transplantation of whole ovaries in animals have yielded encouraging results. However, it has not been possible to perform whole ovary transplantation after cryopreservation in humans due to technical difficulties in cryopreservation [15]. In this study, optimized perfusion following WOCP&TP in a POI rat model simulating female patients with cancer treated with chemotherapy was demonstrated, with the result that ovarian function and natural fertility were well restored. To the best of our knowledge, this paper is the first to report a third generation of offspring obtained after WOCP&TP.

The major technical problem with whole ovary freezing is the increased volume, complexity, and diversity of tissues leading to slow penetration of CPA and increased risk of damage to the cellular structure. Studies showed that perfusion of the ovary via its afferent vessel with CPA increased survival and restoration of ovarian function [16, 17]. However, vascular perfusion, both alone and in combination with cryopreservation, showed deleterious effects on the ovarian stroma and follicles [8, 9, 18]. The mechanism of cellular damage and loss is mainly ice crystal formation. Not only the formation of intracellular ice but also the formation of ice in extracellular locations, particularly in blood vessels, can produce serious injury and prevent organ revascularization during cooling and warming [19]. Therefore, effective perfusion methods need to be explored [20].

Based on the theory of cryobiology, the ideal concentration of CPA that permeates into cells should be linearly increasing, and maintained in balance for a period after reaching the final concentration in order to avoid ice formation. Hence, we designed a new perfusion apparatus that consists of two peristaltic pumps to provide an accurate, controlled concentration of CPA in order to improve efficiency and minimize the detrimental effects of perfusion. We deduced practical data according to the law of mass conservation. The whole ovaries were perfused before cryopreservation in a linearly increasing mode and, after thawing, in a linearly decreasing mode so that the intracellular and intercellular water in the graft organ was removed and substituted. In addition, the flow rate of perfusion was controlled to avoid vascular damage. The procedure of slow freezing and rapid thawing was also performed to reduce ice formation. We found well-preserved general morphology of the follicles and medulla and high rates (77.6%) of follicle survival, although the population of follicles was significantly reduced compared with the fresh group. It may seem paradoxical that there was no significant difference in the number of antral follicles between the two groups, but antral follicles were significantly apoptotic on apoptotic assay. A main reason for this is that water content is much higher in antral follicles, leading to intolerance of cryopreservation; another explanation may be that oocyte cytoplasm staining varied from one slice to another, which influences the analysis [21–23].

It would be useful to prove the efficacy of whole ovary removal and cryopreservation in patients with cancer prior to chemo- or radiotherapy, with subsequent thawing and autotransplantation at an appropriate time to restore ovarian function and fertility. Therefore, a POI rat model induced by CTX was established in order to simulate the procedure. POI rats displayed abnormal estrus cycles, decreased estrus frequency, higher levels of FSH, lower levels of estradiol-17β and progesterone, and contracted ovaries. After 2 weeks of CTX treatment, with recovery of general condition but a continued POI state, the rats underwent orthotopic transplantation of whole ovaries with vascular anastomosis. Hence, allotransplantation but not autotransplantation was established due to the use of a rodent model. However, no immunosuppressor was used because the rats were homogenic. The reason we used rats as an experimental model for research is that they are small and inexpensive animals with a high reproductive efficiency. Moreover, the knowledge of ovarian function in the rat, especially folliculogenesis, is vast [24]. Further studies in autotransplantation could be performed in larger animals, such as sheep.

In the present study, high rates (70%) of restoration of ovarian function were obtained following WOCP&TP. Previous studies in animals showed low rates (< 50%) of restoration of ovarian function despite apparently high rates of post-thaw tissue viability [4, 8, 25–29]. It was first reported in the study by Campbell BK et al. [30] that full (100%) restoration of acute ovarian function was obtained following WOCP&TP of adult sheep ovaries by utilizing optimized cryopreservation and postoperative anticoagulant regimes. They found that 60 min of perfusion with CPA prior to WOCP was optimal for whole sheep ovaries, achieving adequate penetration while minimizing CPA toxicity. In our study, the exposure time of CPA was 20 min for whole rat ovaries, which can be explained by the smaller size of rat ovaries. The results of the present study further support the importance of optimizing the cryopreservation protocol for different types of ovaries prior to transplantation. Therefore, further optimization utilizing other animal ovary models of an appropriate size and/or donated human ovaries is required.

In this study, 14 rats began to recover ovarian function 2 weeks after WOCP&TP, and showed normal ovarian function 4 weeks after WOCP&TP. Previous studies [29, 31] in rats showed that recovery started 10-12 days after transplantation, slightly earlier than in our experiment. The time of full ovarian function recovery was not clearly demonstrated in previous rat studies. While the animals may have functioning ovaries, a certain number of animals may not show regular estrous cycles. In the present study, the 14 rats showed normal fluctuation in hormone levels and normal weight of the transplanted ovaries, indicating full restoration of ovarian functions. The reason that rats with WOCP&TP failed to recover ovarian function may be due to necrosis or atrophy of the ovarian tissue resulting from infection or insufficient blood supply, based on postmortem examination.

High live birth rates (20%) were obtained naturally after WOCP&TP in this study, and the first filial generation of rats gave live birth to second and third generations. All the offspring were alive and normal in appearance, suggesting that the nature of successive generations was unaffected by cryopreservation. This positive finding holds promise for the clinical application of fertility preservation in women who require ovarian cryopreservation. However, further experimentation, such as molecular testing, is required to investigate any influence of cryopreservation on genetic stability. In addition, not all rats that cycled regularly got pregnant. It is likely that adhesions around the transplanted ovary and uterus physically obstructed gamete transport and/or caused ovulatory dysfunction, based on postmortem findings. Further studies are required to resolve this. However, in clinical practice, fertility restoration in women following WOCP&TP need not to be totally reliant on the success of natural ovulation, as it can be supported through the use of established assisted reproduction interventions [30].

In our study, high rates of ovarian follicles were restored by utilizing optimized perfusion before cryopreservation. In addition, high rates of restoration of ovarian function were obtained following WOCP&TP in a CTX-induced POI rat model, and multiple generations of offspring were born with no abnormalities. However, as mentioned before, the present study may be subject to limitations, mainly because of the use of a rodent model. Rat ovaries are much smaller than human ovaries and therefore may better withstand the procedures used. Furthermore, no full restoration of ovarian function was achieved. Further optimization of whole ovaries utilizing other animal models of an appropriate size and/or donated human ovaries is required.

## Conclusions

In conclusion, the results of this study demonstrate that high rates of restoration of ovarian function and natural fertility with multiple generations of offspring can be achieved after WOCP&TP in CTX-induced POI rat models by optimization of CPA penetration during whole ovary perfusion. Moreover, cryopreservation did not affect the nature of successive generations. New evidence has been added concerning the possible development and clinical application of WOCP&TP as a way to preserve and restore fertility of girls and women at risk of POI. However, further animal studies are required to develop optimized cryopreservation protocols for a range of different sized ovaries and to minimize postoperative inflammatory effects, adhesion, and thrombosis before human trials could commence in the application of WOCP&TP.

## Methods

### Experimental animals

Healthy adult female Lewis rats aged 8-10 weeks and weighing 180-200 g were obtained from Bikai Corporation (Shanghai, China). The animals were fed at the Animal Experiment Efficacy Evaluation Center, School of Pharmacy, Fudan University. The animals were housed in groups of 5 per cage and were maintained under standard laboratory conditions (12 h of light, 12 h of dark; 25 °C) for 7-10 days to acclimatize. During acclimatization, standard mouse chow and water were available ad libitum. Vaginal smears were obtained daily. Only rats showing at least two consecutive normal 5-day vaginal estrus cycles were included in the experiments.

### Animal model of POI establishment

Fifty Lewis rats were divided into two groups: the POI group and the control group. To establish animal models of chemotherapy-induced ovarian failure, 25 rats received a loading dose of intraperitoneal CTX 50 mg/kg (Baxter Oncology GmbH, German) followed by a daily intraperitoneal CTX injection of 8 mg/kg for 14 consecutive days. At the same time, 25 rats in the control group received 1 ml of 0.9% sodium chloride. All animals were monitored and weighed daily to observe their condition. Vaginal smears were obtained daily. Blood samples were collected to determine serum levels follicle stimulating hormone (FSH), progesterone, and estradiol-17β levels by enzyme-linked immunosorbent assay kits (Shanghai, China). Rats diagnosed with POI were transplanted after 2 weeks of treatment.

### Ovarian collection

Forty Lewis rats were anaesthetized with sodium pentobarbital (40 mg/kg). With the aid of an operating microscope (GX.SS.22-3, Shanghai Medical Optical Instruments Co., China), the right ovary, fallopian tubes, and the upper third of the right uterus were dissected and branches of their vessels were divided. The ovaries were removed en bloc with their arteries and veins attached, with the ovarian vessels dissected to create short cuffs of the aorta and vena cava. The aorta was cannulated using a 2.5 F (0.75 mm OD) intravenous cannula that was tied securely in place, using 5-0 mersilk ligatures. The ovaries were perfused with heparinized Ringer’s solution via aorta cannulation in vitro. The whole ovary was cut down and subsequently immersed at 4 °C until required. Ovaries were randomly assigned to three groups. In the fresh group (*n* = 5), the ovary was cut from the pedicle and immediately fixed in 4% paraformaldehyde for subsequent analysis. In the conventional group (n = 5), the ovary was perfused in a conventional mode at a rate of 0.35 ml/min with M2 medium containing sucrose as well as an increasing concentrations of 0 M, 1 M, and 1.5 M DMSO for 10 min, respectively, cryopreserved and thawed, then cut from the pedicle and immediately fixed in 4% paraformaldehyde for subsequent analysis. In the optimized group (*n* = 5), the ovary was perfused in the new mode (described below), cryopreserved and thawed, then cut from the pedicle and immediately fixed in 4% paraformaldehyde for subsequent analysis. In the POI group, as donors (*n* = 25), the ovary was perfused in the new mode, cryopreserved and thawed, then transplanted into the POI rats.

### Ovarian perfusion, cryopreservation, and thawing

**Fig. 7:**
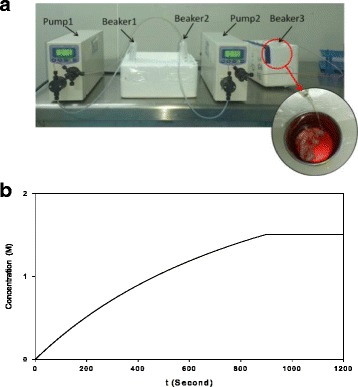
The optimized perfusion apparatus. **a** Illustration of the perfusion apparatus. Arrow 1 and arrow 2 were pulse control pumps. Arrows 3, 4, and 5 were containers. **b** The increasing mode of optimized perfusion. The concentration of dimethyl sulphoxide was increased from 0 M to 1.5 M at the beginning of 15 min and was maintained at 1.5 M for the last 5 min

The apparatus used for perfusion of the whole ovary is newly designed by our research team (Fig. 7). The apparatus mainly consists of two pulse control pumps (SP-12, Ever Seiko Corporation, Japan) with outlet and inlet pipes and three containers. The perfusion speed is controlled accurately by the peristaltic pump of the apparatus. The solution in the first container, which held an M2 medium containing 0.2 M trehalose and 3 M DMSO, was pumped into the second container at a rate of 0.15 ml/min. The solution in the second container, an M2 medium containing 10% fetal bovine serum, was pumped into the third container at a rate of 0.35 ml/min. A vein detained needle (24 G, 0.7 mm × 19 mm, BD, China) at the end of the output pipe was connected to the aortic cannula. The solution in the third container was an M2 medium containing 0.1 M trehalose, 1.5 M DMSO, and 10% fetal bovine serum. The whole ovary with the pedicle in the third container was perfused for 20 min, so that the concentration of DMSO increased from 0 M to 1.5 M in the first 15 min and then maintained at 1.5 M for the last 5 min. The whole ovaries were perfused in a mode of linearly increasing DMSO concentration at a constant speed of 0.35 ml/min for 20 min before cryopreservation. The media was adjusted to a pH of 7.3-7.4 and sterilized using a 0.22 μm syringe filter (Merck Millipore, China) prior to use. All solutions were refrigerated before use and all perfusions were carried out on ice.

After perfusion, the grafts were immersed in cryoprotective solution and transferred to 25-ml Nalgene cryovials. With the aid of a Planer controlled-rate freezer (Kryo 550-16; Planer plc, Middlesex, UK) a slow-freezing cryopreservation protocol was utilized, which involved cooling to − 7 °C at 2 °C/min before manual seeding was performed. Cooling continued at 0.3 °C/min to − 40 °C and subsequently at 10 °C/min to a final temperature of − 140 °C. The cryogenic vials were then plunged into and stored in liquid nitrogen for at least 2 weeks prior to evaluation or transplantation.

Thawing was carried out rapidly by swirling the ovaries in air for 1 min and then in a water bath at 40 °C for 5 min. After being completely thawed, the grafts were transferred to a perfusion tray and immediately reperfused. The CPA was washed out by reversing the concentration gradient at the same flow rate as before. The cannula was then eased from the artery that was then ready for reanastomosis or examination. The grafts were held in preservation solution on ice until transplantation within 15 min.

### Ovarian transplantation

Recipient POI rats were anaesthetized with sodium pentobarbital (40 mg/kg). The transplantation operation was performed according to the methodology described by Wang et al. The aorta and vena cava below the left renal artery were dissected free and prepared for end-to-side anastomosis. Longitudinal incisions of 2-3 mm were cut in the great vessels and flushed with heparinized Ringer’s solution prior to aortic-aorta and veno-venous anastomoses using continuous suturing with 10/0 nylon (Ethicon Inc., USA). Both ovaries, fallopian tubes, and the upper third of the uterus were removed. The uterine segment of the cryopreserved organ was sutured to the corresponding end of the host’s organ with 7/0 synthetic absorbable sterile surgical suture (Ethicon Inc., USA). The average duration of the operation was 1 h and in every case blood was observed to surge back into the organs after reanastomosis. The rats in the control group underwent a sham operation.

### Assessment of transplanted ovarian viability in vivo

Vaginal smears were prepared daily to monitor ovarian activity for more than 2 months after transplantation. Whole blood samples were collected every 2 weeks from the orbit after transplantation. After resting at 25 °C, the blood samples were centrifuged 3000 xg for 15 min to obtain plasma, which was stored at − 20 °C until assay performance. Levels of FSH, estradiol-17β, progesterone, and anti-Müllerian hormone (AMH) were determined by enzyme-linked immunosorbent assay kits (Shanghai, China).

### Assessment of fertility

Healthy adult male Lewis rats aged 10 weeks were obtained from Bikai Corporation (Shanghai, China). The female rats that recovered ovarian function based on examination of ovarian cyclicity were mated. One month after delivery, one rat underwent a second mating. The first filial generation of offspring was mated when they were 8-10 weeks old. In the same way, the second generation of offspring was mated when they were 8-10 weeks old. The number of live births and the physical appearance of the offspring were observed.

### Histological analyses

Rats were sacrificed 8 months after transplantation. The transplanted ovary was dissected and fixed in 4% paraformaldehyde for later histological analysis. After routine paraffin embedding, thin sections (4-5 μm thick) of all ovarian tissue were prepared and stained with hematoxylin and eosin. Within these sections, the number of follicles present were recorded and classified as primordial (oocyte surrounded by a single flat layer of follicle epithelial cells/pregranulosa cells), primary (single layer of cuboidal granulosa cells), secondary (two or more layers of granulose cells, no antrum), or antral (presence of an antrum), similar to previously described methods. Evaluation of the normality of the follicles was based on examination of the integrity of the basement membrane, cellular density, presence or absence of pyknotic bodies, and integrity of the oocyte. Based on these criteria, follicles were classified as morphologically normal or abnormal. In addition, the density of the stromal cells in the ovarian medulla was analyzed under high magnification (× 400).

### Apoptosis analysis

Apoptotic cells were identified with a terminal deoxynucleotidyl transferase-mediated deoxyuridine triphosphate nick end-labeling (TUNEL) kit (Roche Diagnostics, America) and a Bax kit (Abcam Inc., England) according to the manufacturer’s manual. Positive cells appeared as brown granules. Blinded samples were observed under microscopy (Olympus DP73, Japan). Five different regions in each slide were randomly selected and pictured. The numbers of TUNEL-positive cells and Bax-positive cells were calculated as the average of the numerical values using image analysis software Image-Pro Plus Vision 6.0 (Media Cybernetics, America).

### Statistical analyses

Statistical analyses were performed using SPSS 14. All data were expressed as means ± SEM. The Student *t* test was used to determine significant differences between two groups. A one-way analysis of variance with least significant difference tests was used to determine significant differences among the four groups. *P* < 0.05 was considered to be statistically significant.

## Abbreviations

AMH: Anti-Müllerian hormone
CPA: Cryoprotective agents
CTX: Cyclophosphamide
DMSO: Dimethyl sulphoxide
FSH: Follicle stimulating hormone
POI: Premature ovarian insufficiency
TUNEL: Terminal deoxynucleotidyl transferase-mediated deoxyuridine triphosphate nick end-labeling
WOCP&TP: Whole ovary cryopreservation and transplantation
